# The Potential Role of Flavonoids in Ameliorating Diabetic Cardiomyopathy via Alleviation of Cardiac Oxidative Stress, Inflammation and Apoptosis

**DOI:** 10.3390/ijms22105094

**Published:** 2021-05-12

**Authors:** Fatin Farhana Jubaidi, Satirah Zainalabidin, Izatus Shima Taib, Zariyantey Abd Hamid, Siti Balkis Budin

**Affiliations:** 1 Center for Diagnostic, Therapeutic and Investigative Studies, Faculty of Health Sciences, Universiti Kebangsaan Malaysia, Kuala Lumpur 50300, Malaysia; fatinfarhanajubaidi@gmail.com (F.F.J.); izatusshima@ukm.edu.my (I.S.T.); zyantey@ukm.edu.my (Z.A.H.); 2 Center for Toxicology and Health Risk Research, Faculty of Health Sciences, Universiti Kebangsaan Malaysia, Kuala Lumpur 50300, Malaysia; satirah@ukm.edu.my

**Keywords:** diastolic dysfunction, flavone, flavonol, flavanol, isoflavone, systolic dysfunction

## Abstract

Diabetic cardiomyopathy is one of the major mortality risk factors among diabetic patients worldwide. It has been established that most of the cardiac structural and functional alterations in the diabetic cardiomyopathy condition resulted from the hyperglycemia-induced persistent oxidative stress in the heart, resulting in the maladaptive responses of inflammation and apoptosis. Flavonoids, the most abundant phytochemical in plants, have been reported to exhibit diverse therapeutic potential in medicine and other biological activities. Flavonoids have been widely studied for their effects in protecting the heart against diabetes-induced cardiomyopathy. The potential of flavonoids in alleviating diabetic cardiomyopathy is mainly related with their remedial actions as anti-hyperglycemic, antioxidant, anti-inflammatory, and anti-apoptotic agents. In this review, we summarize the latest findings of flavonoid treatments on diabetic cardiomyopathy as well as elucidating the mechanisms involved.

## 1. Introduction

The incidence of diabetes mellitus and its related complications have become a threat to global health and is a major factor to high morbidity and mortality. Leading up to 2045, it is expected that the global population of diabetic patients will rise to up to 700 million [1]. Cardiovascular diseases generally are the main factors that contribute to the high morbidity among diabetic patients [2]. A study on cardiovascular-related hospitalizations has revealed that more than 35% of all hospitalization were patients with diabetes mellitus and that more than 25% are due to diabetic-related cardiovascular complications [3].

Diabetic cardiomyopathy (DCM) can be defined as abnormality in the myocardial structure and function in the absence of other cardiovascular factors, such as artery coronary diseases, hypertension, congenital and valvular heart diseases [4]. DCM is characterized by left ventricular wall hypertrophy with regressed diastolic function at its early development before this condition worsens as extensive cardiac fibrosis and hypertrophy develops, causing further impairment of diastolic dysfunction. The advanced stage of DCM is characterized with the appearance of systolic dysfunction along with the aforementioned cardiac structural and functional disturbances [5]. In a clinical scenario, DCM development could only be detected when the signs and symptoms of regressed cardiac functions appeared, as DCM has reached the late stage (significant diastolic and systolic dysfunction) and heart failure events have already been initiated [6].

To date, there is no specific treatment to treat and limit diabetic cardiomyopathy progression. Diabetic patients are only given therapeutic drugs to manage the hyperglycemic condition (via management with glucose-lowering agents and insulin therapy) and cardiovascular complications (via the administration of beta blockers and angiotensin-converting enzyme inhibitors) [7,8]. However, these medications have specific toxic side effects, and long-term use of insulin therapy causes decreased insulin receptor sensitivity, resulting in insulin resistance and eventually leading to worsening of control conditions [9]. Currently, several new targeted drugs have been developed and have entered the market, such as DPP-4 inhibitors, GLP-1 analogs, and SGLT-2 inhibitors [10]. However, the high cost of these drugs leads to difficulties in meeting the high demand from patients with diabetes and limits the clinical application of these drugs. Therefore, research exploring the alternative treatment to treat and limit the progression of DCM is very much needed.

Flavonoids are the most common occurring plant phenolic compounds [11]. On average, flavonoids are consumed daily from diet at approximately 400 mg/kg aglycone equivalent (AE). They have been reported to exhibit diverse therapeutic potential in medicine and other biological activities [12,13,14]. The potential of flavonoids in alleviating diabetic cardiomyopathy or other cardiovascular complications derived from diabetes have been commonly related with their remedial actions as anti-hyperglycemic [15], anti-hyperlipidemic [16], antioxidant [17], anti-inflammatory [18], and anti-apoptotic [19] agents. Considering the promising potential of flavonoids in combating diabetic cardiomyopathy development, we aimed to review the potential mechanisms that flavonoids and their subclasses target in order to exert their cardioprotective potential.

The literature search was completed using the PubMed database, and relevant keywords (flavonoid; diabetic cardiomyopathy; in vitro; in vivo; inflammation; apoptosis; oxidative stress; fibrosis; hypertrophy; cardiac dysfunction) were used. Based on the search findings, we summarized the articles and integrated the literature search accordingly.

## 2. Diabetic Cardiomyopathy: Understanding the Cardiac Oxidative Stress, Inflammation, and Apoptosis-Related Pathophysiology and Pathogenesis

Rubler et al. [20] gave the earliest clinical description of diabetic cardiomyopathy; they reported post-mortem findings from four diabetic patients whose death were caused by heart failure. In these patients, they noticed that the patients lacked common heart failure risk factors, which are hypertension, myocardial ischemia or any other valvular and congenital heart diseases. Instead, the cardiac failures were established to be caused by the pre-existing diabetes mellitus in the patients. The European Society of Cardiology (ESC) suggested that the diabetic cardiomyopathy condition is related to the cardiac dysfunction observed in diabetic patients in the absence of other cardiac diseases, including coronary artery diseases, uncontrolled hypertension, and congenital and valvular heart diseases [21].

**Figure 1:**
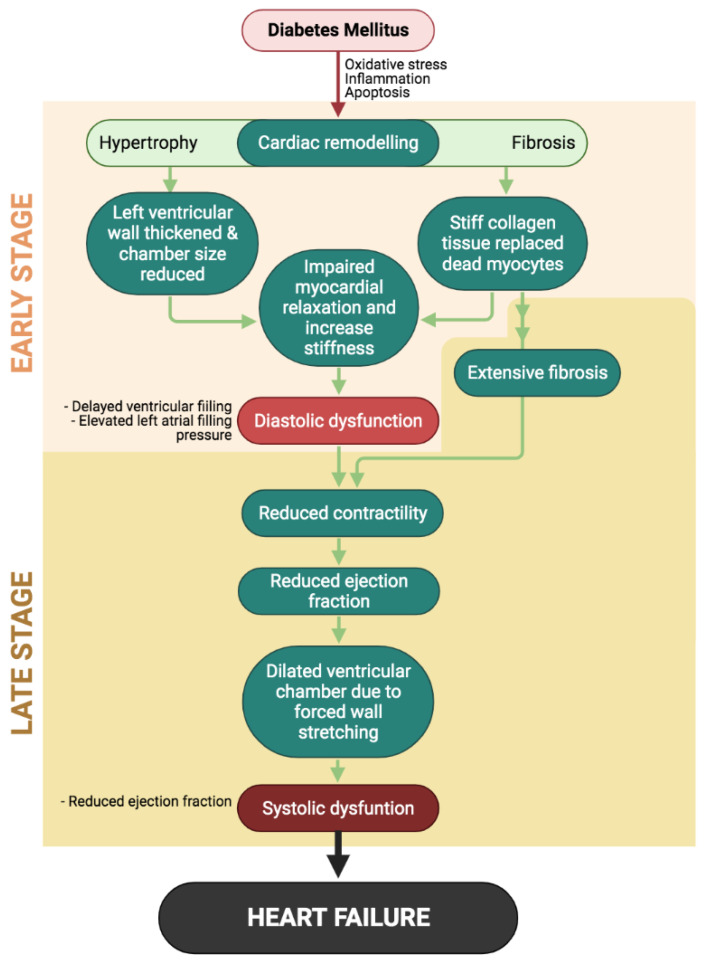
Events in the development and progression of diabetic cardiomyopathy (DCM) initiated by diabetes-induced uncontrolled cardiac remodeling. The early stage of DCM mainly characterized by diastolic dysfunction will progress to late stage as systolic dysfunction appears, eventually leading to heart failure.

As previously mentioned, Paolillo [5] suggested the two stages of diabetic cardiomyopathy; the early stage featuring left ventricular hypertrophy and diastolic dysfunction as the diabetes mellitus progresses; diabetic cardiomyopathy advanced at a late stage with prominent diastolic dysfunction, extensive myocardial fibrosis, systolic dysfunction, and eventually left ventricular dilation (Table 1 and Figure 1). Initially, the asymptomatic left ventricular hypertrophy seen in the early development of DCM resulted from the adaptive response to increased hemodynamic pressure [22], diminished viable cardiomyocyte population [23] and neurohormonal activation [24]. Reduction in cardiomyocyte population is caused by extensive cardiomyocyte death resulting from oxidative damage and apoptosis is induced by chronic hyperglycemia. Due to this, viable cardiomyocytes have to bear the burden to generate enough force in order to pump blood from and into the heart, subsequently causing the cells to enlarge, known as the pathological hypertrophy [25,26].

Aside from cardiac hypertrophy, myocardial fibrosis is one of the main features defining the structural changes in DCM. Fibrosis occurs along with hypertrophy as a cardiac remodeling process to preserve the heart structure. The deposition of fibrotic tissue, including collagen I and collagen III, in the extracellular matrix (ECM) space is to replace the dead cardiomyocytes, and the stiff nature of collagen tissue causes the heart to lose its contractility and flexibility [27]. Gliozzi et al. [28] explained that the downregulation of matrix metallaprotenaise-2 (MMP-2) during the progression of DCM results in collagen deposition in the ECM, leading to the rigid myocardium. The downregulation of MMP-2 was associated with the translocation of N terminal truncated MMP-2 from the cytosol into the mitochondria, leading to mitochondrial dysfunction that enhances cardiac dysfunction. Via synthesis and release of growth factors and cytokines, a hyperglycemia condition also promotes fibroblast proliferation and transdifferentiation of myofibroblasts as well as activating the transcription of extracellular matrix protein [29].

Hyperglycemia, a common trait shared in both Type 1 (insulin dependent) and Type 2 (non-insulin dependent) diabetes mellitus, resulting from impaired insulin signaling, is one of the major etiologies for cardiovascular complications in diabetic patients. Disorder of glycometabolism leads to the persistent hyperglycemia which induces reactive oxygen species (ROS) production in the myocardium via several pathways, including the generation of advanced glycation end products (AGE) as well as activation of polyol, hexosamine, and protein kinase C pathways [30]. Accumulation of ROS further aggravates myocardial oxidative stress, exacerbating dysfunction of mitochondria, which leads to cardiomyocyte deaths and the subsequent cardiac remodeling (cardiac hypertrophy and fibrosis) to preserve the cardiac function and integrity. Uncontrolled and excessive cardiac remodeling further disturbs the ability of the heart to contract and relax efficiently, resulting in the development of diastolic and systolic dysfunctions along with the appearance of prominent cardiac structural changes, both of which are the diagnostic features of diabetic cardiomyopathy [5].

**Figure 2:**
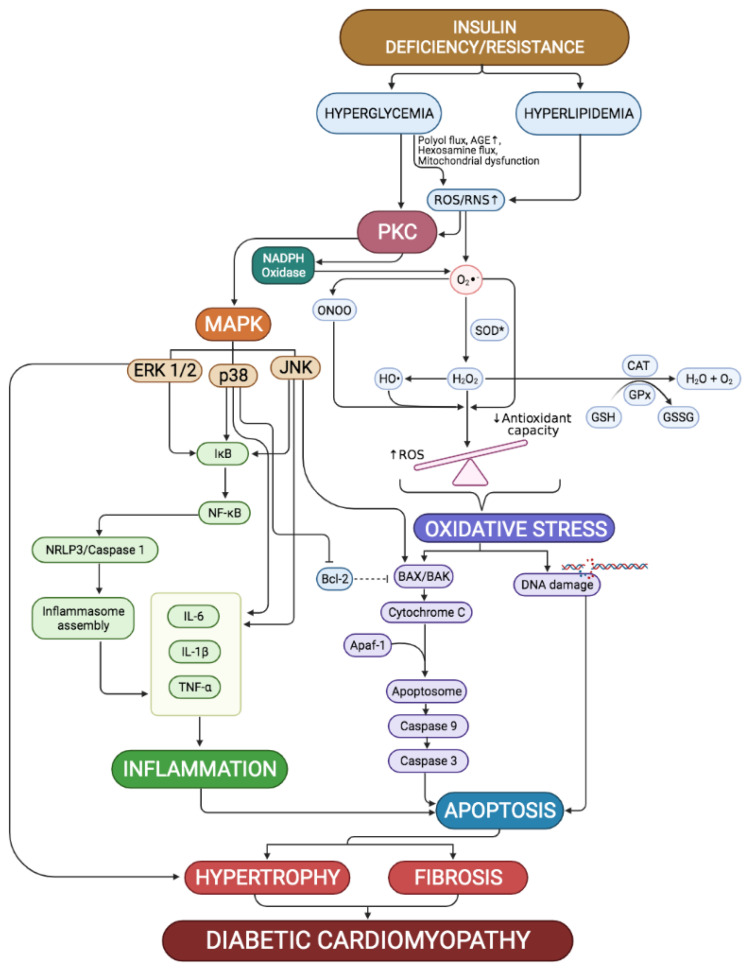
Summary of the mechanisms involved in the pathogenesis of diabetic cardiomyopathy. Hyperglycemia and hyperlipidemia resulting from insulin dysregulation induces excessive production of reactive oxygen and nitrogen species (ROS and RNS), generating oxidative stress in the cardiomyocytes. ROS also activates protein kinase C (PKC) pathways, which induces inflammation and cell apoptosis via mitogen-activated protein kinase (MAPK) pathways. Uncontrolled cardiomyocyte deaths lead to cardiac remodeling by cardiomyocyte hypertrophy and myocardial fibrosis as adaptive mechanisms to preserve the integrity of the heart. This structural alteration causes the heart to functionally regress as both diastolic and systolic dysfunction developed, conditions which define diabetic cardiomyopathy.

Cardiomyocyte death occurs via several pathways including lipotoxicity and production of AGE following high glucose level in the blood. AGE production induces inflammation, infiltration of immune cells, and apoptotic factors [31], as summarized in Figure 2. Replacement of dead cells with fibrosis is aggravated when protein kinase C (PKC) and its downstream cascades and proteins are activated by hyperglycemia. Chronic exposure to a high glucose environment is related to organ and tissue injury including micro- and macrovascular injury, neuropathy, and nephropathy. Hyperglycemia-induced oxidative stress activates several inflammatory and apoptotic pathways that are involved in the development of DCM [32]. Aside from lipid peroxidation and protein carbonylation in the cell membrane of cells, reactive oxygen species (ROS) that are excessively produced could also activate inflammatory and apoptotic signaling pathways in the heart [33]. Moreover, previous reports also mentioned the increased pro-inflammatory cytokines expression in the diabetic hearts of experimental rodent models [34,35]. Moreover, excessive superoxide production that overwhelms the cardiac natural antioxidant defense has been widely discussed to play a significant role in the development of DCM. Furthermore, they overtly trigger various pathways, which leads to the deterioration and alteration of cardiac function and structure; the superoxide itself was found to directly induce cardiomyocyte apoptosis aside from mitochondrial dysfunction [28].

Persistent hyperglycemia not only increases ROS production, but it can also result in the de novo production of diacylglycerol (DAG) via the glycolytic process of glucose [36]. DAG plays a role as an important cellular second messenger and is known to mediate the activation of the PKC signaling pathway. Aside from the DAG trigger, PKC pathways are also directly activated by accumulating ROS. Production of ROS is further enhanced by the activation of mitochondrial NADPH oxidase by PKC. Mediated by the activation of mitogen-activated protein kinases (MAPKs) pathways downstream of PKC signaling pathway, cardiomyocyte inflammation and apoptosis were further exacerbated [37]. While activation of extracellular regulated kinase (ERK) 1/2 pathway is suggested to promote cardiomyocyte survival via its anti-apoptotic effect [38], its activation also further enhances cardiomyocyte hypertrophy, making it a double-edged sword. Augmentation of cardiomyocyte hypertrophy via the activation of p38-MAPK and c-Jun N-terminal kinase (JNK) pathways dull the cell survival effect of ERK 1/2 by increasing the Bax/Bcl-2 apoptotic ratio [39].

### 2.1. Cardiac Oxidative Stress

Oxidative stress is a well-established etiology for various pathologies, including diabetic cardiomyopathy. Oxidative stress results from the redox imbalance in the cell, whereby the endogenous antioxidant defense system is overwhelmed by the accumulation of ROS [40]. Accumulation of ROS and its persistent high level is enhanced by impaired endogenous antioxidant system, leading to diabetes-associated cell inflammatory and apoptotic response, thus contributing to the development and progression of DCM. Mitochondrial NADPH oxidase activation is a major source of cardiac ROS in diabetic conditions, boosted by diabetic-induced mitochondrial dysfunction and activation of PKC pathway [4]. In normal condition, endogenous superoxide dismutase (SOD) will catalyze the dismutation of superoxide radicals into hydrogen peroxide (H_2_O_2_). H_2_O_2_ will then break down into a water and oxygen molecule by catalase and the indirect action of glutathione peroxidase [4]. Production of advanced glycation end products (AGE) by non-enzymatic glycosylation of protein and lipid could increase the production of ROS. AGE would bind to its receptor on cell surface, the receptor for AGE (RAGE), inducing inflammatory response and apoptosis of the cells [41].

Hyperglycemia-induced oxidative stress has been heavily implicated as the key role in the development and progression of cardiac dysfunction in diabetic patients. Via the elevation in NADPH oxidase activity, which results in the excessive production of ROS, cardiac function was compromised as this process promotes Ca^2+^ intake into the cardiomyocytes. Excessive ROS production was also found to induce cardiomyocyte apoptosis, mediated by overexpression of 18 kDa translocator protein (TSPO) and voltage-dependent anion-selective channel 1 (VDAC1) that is involved in the formation of mitochondrial transition pores, resulting in mitochondrial dysfunction and cardiomyocyte apoptosis [27].

The activation of nuclear erythroid 2-related factor 2 (Nrf2) has been associated with a cardioprotective effect via the regulation of cellular antioxidant productions. Upregulation of Nrf2 has been shown to improve cardiac oxidative stress, making it an ideal target for cardiac adjuvant therapy, especially in preventing hyperglycemia-induced development of DCM, which is closely related to uncontrolled oxidative stress [42]. Normally, Nrf2 is bound to Kelch-like ECH-associated protein (Keap1) and stays in the cystosol. Upon activation, Keap1 dissociates from Nrf2, allowing it to translocate into the nucleus and bind to its promoter side. This results in the transcription of genes encoding for endogenous antioxidants (SOD, catalase, glutathione peroxidase) and phase II antioxidant enzymes (heme oxygenase-1, NADPH dehydrogenase and γ-glutamylcysteine synthtase), thus alleviating oxidative stress [43]. As oxidative stress brings about most cardiac complications in the diabetic condition, the Nrf2 pathway serves as one of the best targets in treating DCM.

### 2.2. Cardiac Inflammation

Cardiac inflammation has been reportedly responsible in DCM development [44]. Inflammation is considered as an adaptive response to restore cellular homeostasis when stress is being exerted on cells [34]. However, when the stress that is being exerted is persistent, such as in diabetes, it does not take long for this adaptive response to turn maladaptive and exert damaging effects instead. Studies showed that pro-inflammatory cytokines including tumor necrosis factor-α (TNF-α), interleukin-1β (IL-1β) and IL-6 all play a major role in the development of cardiomyocyte hypertrophy [45,46]. Persistent hyperglycemia was found to induce the expression of these cytokines in the heart by activating the MAPK pathways (JNK and p38-MAPK), causing cardiac damage, which further aggravates the infiltration and accumulation of leukocytes onto the affected site [47]. On top of that, it has been implicated that the downregulation of sirtuin-1 (SIRT-1) activity induced by persistent hyperglycemia condition and high insulin environment also plays a significant role in the development of diabetic cardiac inflammation [48].

Cardiac inflammation is also enhanced by increased lipid level in the blood, which in turn would activate toll-like receptor-4 (TLR-4) and aggravate cardiac inflammation via the NF-κB pathway [49]. Both hyperlipidemia and hyperglycemia enhance cardiac inflammation by activating the PKC/MAPK pathways. Degradation of IκB via both p-38 and JNK pathways activates NF-κB [50]. Oxidative stress is also further exacerbated by persistent hyperglycemia and hyperlipidemia, in which PKC activation also plays a role by activating mitochondrial NADPH oxidase [51]. Inhibition of Nrf-2 by ERK 1/2 exaggerates oxidative stress, which in turn activates NF-κB [52]. These evidence shows that via activating NF-κB, which promotes the production of pro-inflammatory cytokines and causes hyperglycemia and hyperlipidemia, which are able to induce cardiac inflammation.

### 2.3. Cardiomyocyte Apoptosis

Apoptosis, also known as programmed cell death, is the quickest form of cell death [53]. Cardiomyocyte apoptosis can occur when adaptive responses to return cardiac homeostasis and stressor-reducing attempts have all failed. Chen et al. [54] closely correlates persistent hyperglycemia with the eventual cardiomyocyte apoptosis. In other organs, such as skin and liver, apoptosis is an important process to maintain the organ and tissue homeostasis. However, in organs in which cells have very limited regeneration ability, cell death by apoptosis is rather damaging, as the tissue is unable to completely regenerate back to its original condition [54]. Extensive cardiomyocyte apoptosis results in the loss of contractile units of the heart. This results in substantial cardiac hypertrophy and fibrosis, consequently reducing the heart’s ability to contract and relax as normal, which leads to the development of diastolic and systolic dysfunction, eventually leading to heart failure if left without intervention [5].

Both in vivo and clinical studies have proven that cardiac apoptosis plays a major role in the regressed function of the heart in diabetic conditions, mainly induced and derived from a high glucose level [55,56,57]. Caspases are a family of inactive proenzymes that play an important role in apoptosis. Caspases are being activated by other procaspases, depending on the specific activity. This results in a proteolytic cascade that promotes cell apoptosis, which results in permanent cell death [58]. Cell apoptosis occurs via two pathways, the extrinsic and intrinsic pathways, and both may occur concurrently. The extrinsic pathway is also known as the death receptor pathway, as it involves the binding of a stressor onto its receptor on the cell. Examples of cell death inducers are tumor necrosis factor α (TNF-α) and Fas ligands. Their binding activates caspase 8 and caspase 9, which in turn activate the executioner caspases (caspase 3, caspase 6, and caspase 7), resulting in the initiation of apoptosis [53]. The intrinsic pathway, also known as the mitochondrial apoptotic pathway, involves the dysfunction of mitochondria. By the intrinsic pathway, a pore is formed on the mitochondrial membrane by B-cell lymphoma (Bcl-2) protein family members, Bax and Bak, which extend across both inner and outer membranes of the mitochondria. This permits the mitochondrial cytochrome c to be released into the cystosolic compartments, in which interaction with apoptosis protease activating factor-1 (Apaf-1) then forms a complex known as apoptosome. Apoptosomes assist in the cleavage and activation of the downstream caspases, and eventually lead to cell death [54].

Increased apoptotic cell death in the heart of type 1 and type 2 diabetic patients and experimental animal models has been reported in previous study [59]. An experimental study revealed that there was a significant increase in caspase 3 cleavage, caspase 3/7 activities and chromatin fragmentation and increased apoptosis in the heart of diabetic mice [60]. Increased activity of caspase 9, the mitochondrial pathway mediator, was observed in the hearts of diabetic patients [61]. Zhao et al. [62] demonstrated the disturbances in pro- and anti-apoptotic signaling proteins in diabetic myocardial cells. Wang et al. [63] demonstrated an increased rate of apoptosis in the hearts of diabetic mice through a dramatic increase in the Bax/Bcl-2 ratio and the caspase 3 and 9 expression.

## 3. Flavonoid

**Figure 3:**
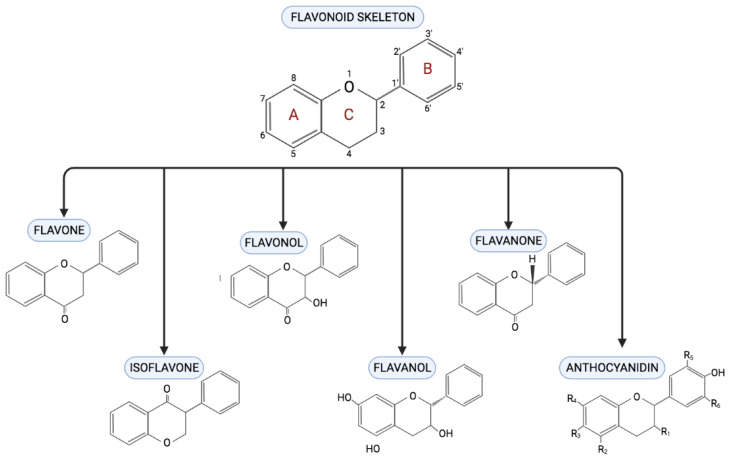
Chemical structures of different classes of flavonols. All flavanols classes share the same chemical bone, comprising two benzene rings (A and B) linked by a heterocyclic ring. Each flavonols are grouped into their classes based on the connection between the B and C rings as well as the hydoxylation and glycosylation patterns of the three main rings.

With at least 6000 compounds being identified, flavonoids are one of the major and largest group of plant secondary metabolites. They are widely found in fruits and vegetables. In general, flavonoid is structurally made up of two benzene rings (A and B), which are linked by a heterocyclic ring containing oxygen (C). As mentioned before, depending on the variant in the chemical structure, flavonoids are differentiated into six different classes [64]. The classes are (1) flavones, (2) flavonol, (3) flavanol, (4) flavanone, (5) isoflavone, and (6) anthocyanidin. As shown in Figure 3, the difference varies because of the connection between the B and C rings, the structure of B ring, and the hydroxylation and glycosylation patterns of the three main rings [65]. Most flavonoids exist in the form of glycosides or carbohydrate groups in plants and partly exist in free form. A majority of plants contain flavonoids, which play an essential role in plant growth, development, and flowering and even have antibacterial and disease prevention effects [66]. Flavonoid use in traditional medicine predates modern medicine, and their medicinal values are mainly because of their potent antioxidant properties. Zhang et al. [67] reported that puerarin, an isoflavone derived from *Radix puerariae* root, was used traditionally as anti-diabetic agents. As summarized in Table 2, flavonoids have been shown to exert protective effects against diabetic cardiomyopathy.

Preparation and processing of food may decrease flavonoid levels depending on the methods used. For example, in a recent study, orange juices were found to contain 81–200 mg/L soluble flavanones, while its content in the juice cloud concentrate was 206–644 mg/L, which suggest that the flavanones are concentrated in the juice cloud concentrate during processing and storage [86]. Accurate estimation of the average dietary intake of flavonoids is difficult, because of the wide varieties of available flavonoids and the extensive distribution in various plants and also the diverse consumption in humans. Recently, there has been an upsurge of interest in the therapeutic potential of medicinal plants, which might be due to their extract being rich in health-beneficial flavonoids.

Flavones can be found in many types of food, especially tea leaves and herbs [87]. They have a double bond in between the C2 and C3 of the flavonoid backbone and absence of any substitution on the C3 position [88]. Among commonly found flavones are nobiletin, baicalein, diosmin, apigenin, luteolin and fortunellin. Flavones are widely studied for their anti-oxidative activities and ability to scavenge ROS [89]. Apigenin, a type of flavone, was found to be able to protect endothelial tissues from inflammatory damage and preserve mitochondrial function [90]. Flavones also exhibited potent anti-inflammatory properties, contributed by regulating the toll-like receptor (TLR)/NF-κB pathway that is responsible for the expression of pro-inflammatory expressions [89].

Flavonols are the most abundantly found flavonoid in nature. They are characterized by the presence of a hydroxyl group in the C3 position (Figure 3), and the majority of flavonols have additional hydroxyl groups in the C5 and C7 positions [91]. Fisetin, kaempferol, quercetin, and myricetin are the most frequently found flavonols in plants, mainly in the leaves and outer parts of the plants (such as peels) [91]. Plants that are rich in flavonols are lettuce, tomatoes, grapes, onions and berries. Flavonols are always associated with their cardiovascular protective effect, mostly contributed by its potent antioxidant potential [66]. Flavonol-rich extracts of ginkgo were found to be able to provide protection against myocardial ischemia reperfusion injury in a rat model [72].

Flavanols, also referred to as flavan-3-ols or catechins, are 3-hydroxy derivatives of flavanones. Different from other flavonoids, flavanols do not have a double bond in between C2 and C3 [66]. They can be found abundantly in apples, blueberries, peaches, and bananas as well as tea leaves. Tea extracts and pure flavanols has been reported to have antioxidant, antiviral, antibacterial, and anti-cancer activities and to decrease blood pressure as well as blood glucose level. Lipid metabolism studies have revealed that tea extracts and individual flavanols lower triacylglycerol and total cholesterol concentrations and inhibit hepatic and body fat accumulation [92].

Isoflavones, another subclass of flavonoids, are often known as phytoestrogen, as they are able to bind on estrogen receptors [93]. They are chemically differed from other flavonoids in that they have hydroxyl groups in the C7 and C4 positions and also differ in the position of the B ring in C3 [63]. Isoflavones are mainly found in legumes, especially soybeans [94]. Genistein, daidzein, and glycetein are among the most commonly found isoflavones in soy, being the most widely studied isoflavones source. Genistein was found to be able to induce alterations in hormonal and metabolic pathways, influencing the affected pathogenesis of the disease [95].

Flavanones, mainly found in citrus fruits, have a dihydropyrone ring (C) between the two aromatic rings (A and B) [66]. They differ structurally from flavones and flavanols, which both are also found widely in citrus fruits, whereby flavanones have chiral carbon atom on the C2 position and absence of any substitution at the C3 position of the C ring [96]. The most frequently found flavanones are hesperetin and its aglycone derivative hesperedin, nanringenin, and taxifolin. Flavanone is mostly consumed from the consumption of citrus fruits and juices sourced from oranges, grapefruits, lemons, bergamots, tangerines, and kumquats. The bitter taste in the citrus peels is contributed by the rich flavanones in this part of the fruits [66]. Flavanones exert their health benefit effects via their potent antioxidant, anti-inflammatory, and lipid-lowering properties [96].

Anthocyanidins, mostly known as anthocyanins, are pigment compounds in plant that contribute to their colors. They occur mainly on the fruit peel of various fruits including grapes, raspberries, blackberries, and cranberries. The color of the anthocyanin depends on the pH and also by methylation or acylation at the hydroxyl groups on the A and B rings [97]. Among mostly studied anthocyanidins are cyanidin, delphinidin, malvidin, and peonidin [66]. The positively charged oxygen atom in the anthocyandin molecule makes it a potent and distinct hydrogen-donating antioxidant [98].

## 4. Therapeutic Role of Flavonoid in Inhibiting DCM Development

### 4.1. Flavones

Reports have shown that flavones could protect the heart against cardiac dysfunction and development of diabetic cardiomyopathy by exerting its action on various mechanisms. Nobiletin, mainly found in citrus peels, were found to be able to alleviate the development diabetic cardiomyopathy via inhibition of cardiomyocyte apoptosis, oxidative stress, and inflammation in rat model when given 1 week after induction of type 1 diabetes mellitus [77]. Alleviation of oxidative stress by nobiletin was via inhibition of phosphorylation of JNK and p38 MAPKs, which in turn downregulate executioner caspase 3 and preventing cardiomyocyte apoptosis. On top of that, nobiletin also suppressed the generation of a superoxide by NADPH oxidase by downregulating expression of NADPH oxidase isoforms [77]. Meanwhile, baicalein was also found to exert the same antioxidative and anti-apoptotic effects, mainly via inhibition of PI3K/Akt pathway in diabetic rats’ hearts [73]. The same antioxidative protection can be seen exerted by fortunellin in high fructose-fed diabetic mice, whereby it was found to increase endogenous antioxidants production (SOD, GSH, CAT, and heme oxygenase-1) by the upregulation of Nrf2. Inhibition of Keap-1 expression was also induced by fortunellin, which in turn upregulates Nrf2, thus preventing diabetic heart injury [74]. Dudylina et al. [99] reported that rutin was able to remove even more superoxide anions compared to flavonol quercetin. They suggested that this is mainly due to the flavone having 2 hydroxyl groups on its B-ring, allowing them to scavenge superoxides more potently. The ability of rutin to scavenge superoxides exhibits the potential in limiting cardiac oxidative damage as well as preventing the excessive trigger of other pathologic pathways that lead to the progression of DCM.

Cardiac inflammation in diabetic animal models was also controlled by flavones, whereby nobiletin, baicalein, and fortunellin were found to be able to reduce the release of anti-inflammatory cytokines (TNF-α, IL-1β, IL-6, and IL-8) [73,74,77]. In a mouse model, fortunellin was found to reduce the phosphorylation of IκB, preventing it to degrade; thus, it inhibits the activation of NF-κB [74,100]. On top of that, diabetic-induced cardiac remodeling by fibrosis and hypertrophy was halted by diosmin and nobiletin treatment to diabetic rats [76,77]. Expression of cardiac hypertrophic and fibrotic markers TGF-β1, CTGF, fibronectin, and collagen I were all significantly reduced upon treatment with both flavones. Interestingly, treatment of diosmin in diabetic rats also seems to improve signaling sensitivity of insulin, which was believed to be caused by improvement in protein metabolism and alteration in muscle and liver glycogen [76].

### 4.2. Flavonols

Similar to flavones, flavonols also control DCM development and progression via their ability to control oxidative stress, inflammation, and apoptosis and the subsequent cardiac remodeling. Persistent hyperglycemia in diabetic patients has been previously reported to be the main trigger for these maladaptive processes [4]. Fisetin, a flavonol abundantly found in fruits and vegetables such as strawberry and cucumber, was reportedly able to lower blood glucose demonstrated by the reduction of the percentage of HbA1c in a diabetic rat model [83]. Feng et al. [80] also demonstrated that kaempferol treatment to diabetic rats was able to reduce the blood glucose level. This glucose-lowering effect of these flavonols was suggested to be contributed by their ability to improve insulin release via their antioxidative effects on pancreatic β-cells, which protect them against further hyperglycemia-induced destruction in type 1 diabetes mellitus [101].

Due to the large number of hydroxyl groups in its chemical structure, quercetin effectively remove superoxide anions produced by mitochondria [99]. Quercetin also reportedly improves nitric oxide bioavailability, thus reducing mitochondrial superoxide production [102]. The upregulation and activation of Nrf2 by flavonols further alleviates oxidative stress in the diabetic heart, thus protecting it against subsequent cardiomyocyte apoptosis, which persistent oxidative stress triggers [80,81,82,83,101,103]. Nrf2 will detach from Keap1 when the latter is S-nitrosylated, allowing it to translocate to the nucleus and bind to its promoter on the DNA. The binding of Nrf2 to its promoter ARE then allows for the upregulation of antioxidant genes, phase 2 detoxification enzymes (heme oxygenase-1, gamma glytamylcystein synthetase, and NADPH dehydrogenase) [104]. Moreover, inhibition of xanthine oxidase by flavonols also protects the heart against oxidative damage, as seen in treatment of rutin to diabetic rats. Inhibition of xanthine oxidase results in the inhibition of inducible nitric oxide synthase (iNOS) activity, generation of AGE, and lipid peroxidation [75]. Flavonol fisetin, kaempferol, and quercetin was also found to improve dyslipidemia in rat models, with a significant increase in the total cholesterol/high density lipoprotein ratio [74,83]. As hyperlipidemia has been associated with alterations of cardiac functions in diabetic conditions, especially by ROS production and lipid accumulation [105], the ability of flavonoids to improve the lipid profile showed that they could reduce cardiovascular risk in diabetic patients.

As inflammation is well correlated with the development of DCM, the ability of flavonols to inhibit this maladapative response induced by hyperglycemia further explains its cardioprotective potential. Treatment of fisetin flavonol to streptozotocin-induced diabetic rats was reportedly able to reduce the production of pro-inflammatory cytokines such as TNF-α, IL-1β, and IL-6 in the hearts of streptozotocin-induced rats [83]. Controlling cardiac inflammation is crucial, as persistent high levels of inflammatory cytokines would further worsen cardiac remodeling via TNF-α’s action on converting resident fibroblast into myofibroblasts, aside from extensive cardiomyocyte death, which is also promoted by TNF-α [106]. Flavonols are also reportedly able to inhibit the phosphorylation of IκB, which in normal conditions would be bound to NF-κB to inactivate it, thus preventing the activation of NF-κB and the subsequent damaging effects it induces [83,107].

Cardiac remodeling events in the diabetic heart is further exacerbated by extensive cardiomyocyte death induced by diabetic conditions. Flavonols treatment was shown to be able to control cardiomyocyte apoptosis [75,80,81,82,83,101,102,103,104]. Both fisetin and kaempferol treatments reportedly downregulated Bax, caspase 9, and caspase 3 expression as well as increased anti-apoptotic protein Bcl-2 in the treated diabetic rats’ hearts [80,83]. In addition, kaempferol was found to downregulate the activation of apoptosis signal-regulating kinase 1 (ASK1) in the hearts of diabetic mice, which mediates inflammation and apoptosis via the activation of NF-κB [99]. Downregulation of ASK1 and its related components further reduces the expression of apoptotic and inflammatory related proteins. Apoptosis of cardiomyocyctes in diabetic conditions is also further aggravated by the activation of mitogen-activated protein kinase (MAPKs) via p-38 MAPK pathways. Flavonol treatment was found to be able to inhibit p-38 MAPK and activate ERK 1/2 pathways, which further improves cell synthesis [79].

While cardiac remodeling comprises events that aim to preserve cardiac function of integrity, uncontrolled and excessive remodeling would result in further decline in cardiac function. Cardiac hypertrophy has been previously reported to be related to the over-activation of MAPK signaling pathways [108]. Kaempferol was found to be able to prevent the phosphorylation of ASK1, which is also the upstream regulator for both JNK and p-38 MAPK [80,109], thus protecting the heart against cardiomyocyte hypertrophy.

### 4.3. Flavanols

Flavanols were found to be able to improve oxidative stress by increasing the phosphorylation of SIRT-1 [70]. Phosphorylation of SIRT-1 will deacytelate forkhead box protein O1 (FOXO1) and perixosome proliferator-activated receptor gamma coactivator (PGC)1-alpha, forming a complex that translocates into the nucleus and binds to the DNA to activate the transcription of SOD2 and catalase, thus increasing their production [70]. Alleviation of accumulated ROS was also found to be exerted by flavanol by the upregulation of phase II antioxidative enzymes, including heme oxygenase-1 (HO-1), NADPH dehydrogenase (NQO1), and manganese SOD (MnSOD) in diabetic rat’s hearts [110]. Both epicatechin and epigallocatechin were found to be able to directly scavenge ROS, thus protecting against direct cardiac oxidative damage in rat models [69]. As hyperglycemia directly causes oxidative stress, the ability of flavanol to exert an anti-diabetic effect by reducing blood glucose level further shows their protection against cardiac oxidative stress in rats [69]. Epigallocatechin was found to be able to inhibit the sodium-dependent glucose transporter 1 (SGLT1) activity, which would prevent systemic glucose uptake [69,111].

Flavanols treatment to diabetic rats is reportedly able to reduce serum level of pro-inflammatory cytokines, which are all involved in the cardiac function and structure derangement in diabetes mellitus [107]. In addition, cardiomyocyte death via apoptosis was also prevented by flavanol treatment, whereby increased Bcl-2 in cardiac tissue was observed following epigallocatechin treatment, thus preventing the mitochondrial release of cytochrome c and the subsequent activation of caspase 9 and the executioner caspase 3 [107]. In another study, epicatechin-rich cocoa polyphenol extract was found to be able to reduce cardiac mitochondrial superoxide production [112]. The reduction in superoxide production is linked to the increased expression of endothelial nitric oxide synthase (eNOS), which restores the bioavailability of nitric oxide. Nitric oxide alleviates mitochondrial superoxide by post-translational modification [113], and in return, ameliorating cardiac oxidative stress.

### 4.4. Isoflavones

Similar to other classes of flavonoids, isoflavones are also able to improve cardiac oxidative stress conditions, as seen in diabetic rats after given treatments of isoflavones genistein and puerarin [85,114]. By promoting the production of endogenous antioxidants, genistein was found to be able to reduce cardiac oxidative damage, reflected by the reduction in cardiac malondialdehyde level compared to untreated diabetic rats [85]. Activation of cardiac Nrf2 again seems to be a common target for most flavonoids as the same activation was also seen upon isoflavone treatments [85,115].

Puerarin, an isoflavone commonly found in legumes and soy, was found to be able to inhibit the signaling of NF-κB, thus impeding cardiac inflammatory response both in vivo and in vitro [115]. Reduction in the expression of pro-inflammatory cytokines further support this finding. It was suggested that upregulated MAPK signaling pathways play an important role in the activation of NF-κB in diabetic conditions. Different structural components are responsible for the activities of isoflavones; for example, the hydroxyl group at position 7 is required for SIRT1 activation, which is a protein deacetylase that regulates metabolism, stress responses, and aging processes [116], while the hydroxyl groups at position 5 block SIRT1 activation, and the loss of the phenyl ring at position 3 or the 4′-hydroxy or -methoxy substituent blocks increases SIRT1 expression [117].

### 4.5. Flavanone

Flavanone use in diabetic cardiomyopathy study has not yet widely explored; however, we can predict the outcome of its treatment by referring to studies made on its effects on other cardiac disease models. For example, naringin, a flavanone mostly found in citrus fruits, exhibited potent antioxidant effects in fructose-treated cells [118]. By modulation of AMP-activated serine/threonine protein kinase-mammalian target of rapamycin (AMPK-mTOR) signaling pathway, naringin inhibited ROS production and cardiomyocyte hypertrophy significantly. Naringin treatment was also able to suppress cardiomyocyte apoptosis, reflected in the significant decreased of caspase 9 and caspase 3 gene expressions in both in vivo and in vitro models [118], which are both more pronounced in non-treated high fructose-exposed H9c2 cells.

Citrus flavanones were also found to modulate the JNK/p38 MAPK pathway, which has been previously reported to play a significant role in the development of DCM [4,17], especially via the promotion of cardiomyocytes inflammation and apoptosis. Musolino et al. [17] reported that via this pathway, bergamot polyphenols, which are rich in flavanone naringin and hesperidin, were able to ameliorate insulin sensitivity along with their potent antioxidative properties, further supporting their therapeutic use for the management of DCM [119,120]. Hesperidin and its aglycone derivative hesperetin are both found abundantly in citrus fruits and showed to be able to protect the heart against doxorubicin-induced oxidative stress by improving cardiac antioxidants levels [121]. Hesperetin exerts anti-apoptotic effects by reducing the activation of p38 MAPK and expression of caspase 3 in the LPS-treated cardiomyocytes [122].

### 4.6. Anthocyanidin

Similar to flavanone, studies of anthocyanidin effects on diabetic cardiomyopathy are not yet widely explored. Only a handful of studies have been completed on its treatment in diabetic cardiomyopathy conditions. As a pigment compound, anthocyanidins are found more frequently in flowers compared to fruits [97]. Treatment of roselle calyx extract, which is reportedly rich in cyanidin-3-glucoside and delphinidin, was shown to improve cardiac dysfunctions in diabetic rats [123,124,125]. Ex vivo treatment of this extract was also able to improve cardiac contraction and relaxation in an ex vivo cardiac function study [126]. Moreover, anthocyanin-rich purple rice extract treatment to diabetic rats was also found to be able to protect the heart against developing cardiac hypertrophy and fibrosis, thus protecting them against developing diabetic cardiomyopathy [68]. However, the pathways on which anthocyanidins acted to exert these cardioprotective effects have not yet been studied.

## 5. Flavonoids in Clinical Studies

The study of flavonoids in clinical settings has not yet been widely explored, especially for isolated flavonoid compounds. Instead, known clinical studies of flavonoid effects on health have mostly focused on the consumption of extracts or fruits that are known to be rich with flavonoids or certain isolated compounds. For example, Curtis and his team [127] studied the effect of blueberry consumption on cardiovascular diseases risk among Type 2 diabetic patients. Blueberries are known to be rich in anthocyanins. They reported that daily intake of one cup of blueberries was able to reduce diabetic cardiovascular disease risk by 12 to 15%, although the consumption did not affect the insulin sensitivity. Macarro et al. [128] studied the benefits of combination of citrus fruits (grapefruit, bitter orange, and olive) that are known to be rich in flavones, on reducing the cardiovascular risk in healthy individuals. They found that the intake of flavone-rich citrus fruits was able to significantly reduce cardiovascular diseases risk. In another study, Curtis and colleagues [129] found that chronic consumption of dietary flavonoids (for three years) was able to reduce the cardiovascular risk in Type 2 diabetes post-menopausal women.

Treating and limiting the progression of DCM has been proven to be tricky due to its nature; multiple mechanisms are being triggered simultaneously in diabetic conditions, leading to the speedy deterioration of cardiac functions. Uncontrolled oxidative stress, apoptosis, and inflammation are all proven to be prompted by persistent hyperglycemic conditions. This is why, presently, controlling optimum or healthy blood glucose level either with the assistance of pharmacological drugs or by the control of diet have been the golden standard in the management of DCM [130]. In that regard, clinical studies have shown that the administration of citrus flavonoids to prediabetic subjects was able to reduce glucose level as well as systemic inflammation and oxidative stress. The 12-week treatment was also found to be able to reverse prediabetic conditions [131]. Similar findings were replicated in obese patients given with bergamot polyphenol-rich extract, with additional favorable insulin control and increased insulin sensitivity following a 90-day treatment [132,133]. Treatment of silymarin, of which the main constituent is flavonoid silibinin, to T2DM patients was found to be able to improve the glycemic index and lipid profile significantly [134]. On top of that, purified anthocyanin treatment was reportedly able to reduce cardiometabolic risk in prediabetic and diabetic subjects, exhibited by the promising glycemic control and improvement of lipid profile [135]. Apple polyphenol treatment for 12 weeks was also found to be able to ameliorate hyperglycemia conditions in prediabetic subjects [136]. Reduction of cardiometabolic risk was also shown following treatment of epicatechin to subjects with hypertriglyceridemia, shown by a significant reduction in the glucose level, lipid profile, and systemic inflammation [137]. The findings of these clinical studies showed the promising potential of flavonoids to be used as cardiovascular disease management, especially DCM, either by cardioprotective properties or anti-hyperglycemic effects.

While some flavonoids share similar mechanisms of action in limiting the progression of DCM, other flavonoids may affect different routes instead. For example, fortunellin inhibits inflammation by inhibiting NF-κB activity [62] and the combination of this compound with fisetin would allow the control of DCM by limiting both cardiac inflammation and cardiac injury via the alleviation of oxidative damage [83]. Theoretically, the combination of flavonoids that exert protective effects via distinct pathways would be most advantageous. Studies have shown that the combination of the flavonoids apigenin and curcumin was found to be able to prevent the progression of cervical cancer cells via an anti-tumor effect on the cancer cells contributed by their synergistic effects [138]. In addition, chrysin and kaempferol combination was found to be able to limit in vitro inflammation by synergistically modulating the TNF-α pathway [139]. These findings, indeed, show that there is a potential for even more effective therapeutic effects when flavonoids are used in combination. However, presently, there is a scarcity of research focusing on this prospect. To the best of our knowledge, research that uses a combination of flavonoids on a DCM model is still lacking. With respect to the usage of flavonoids combination for therapy, there is also a possible contrainteraction between different flavonoids when used in combination, as well as a possible contrainteraction when flavonoids are taken with pharmacological drugs [140]. This unascertained possibility needs to be further studied.

## 6. Conclusions

**Figure 4:**
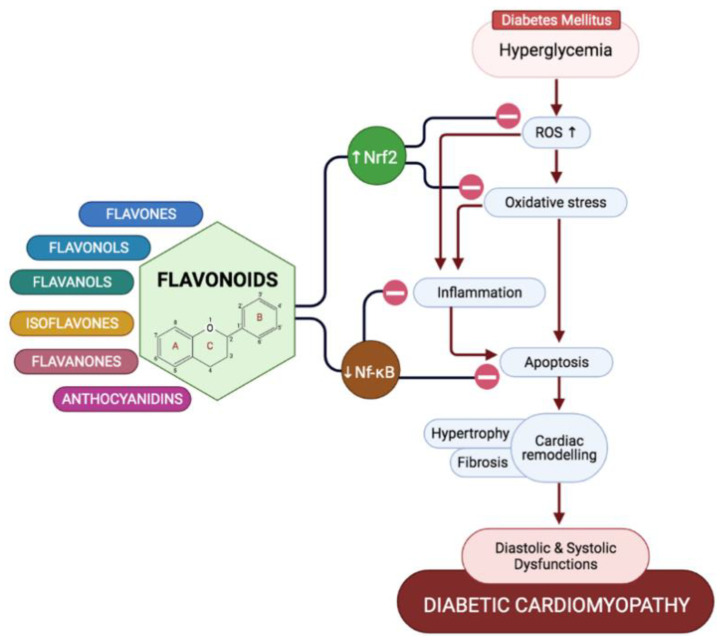
Potential of flavonoids in ameliorating diabetic cardiomyopathy, derived from their ability to combat diabetes-induced oxidative stress, inflammation, and apoptosis, which all play key roles in the structural and functional alterations in diabetic cardiomyopathy.

From our review, it is clear that hyperglycemia-induced persistent oxidative stress exerts the worst effects on the cardiac function in diabetic cardiac oxidative stress, which not only causes direct oxidative damage on the cardiac tissue, but it also propagates cardiac inflammation and apoptosis as adaptive responses, which quickly turns maladaptive when the hyperglycemic condition is not managed, causing a progression of diabetic cardiomyopathy towards an advanced stage. Flavonoid treatments from all classes were shown to be beneficial towards treating and limiting the progression of diabetic cardiomyopathy by mediating hyperglycemia-induced cardiac oxidative stress, inflammation, and apoptosis (Figure 4). All flavonoids seem to share the same effects and targets on alleviating oxidative stress, whereby they were all proven to upregulate the Nrf2 pathway by increasing the endogenous antioxidants levels, assisting the heart to combat the hyperglycemia-induced oxidative stress. They were also shown to inhibit NF-κB pathways in inducing cardiac inflammation. These similar effects by different flavonoids might be due to them sharing a similar chemical backbone. Pre-clinical studies on how flavonoids are able to improve regressed cardiac function in diabetic conditions is very promising for the management of DCM; however, clinical testing on their effects in humans is still lacking and not widely conducted.

## Figures and Tables

**Table 1:** Clinical stages of diabetic cardiomyopathy according to Paolillo et al. [5].

Diabetic Cardiomyopathy Stage	Clinical Findings
Early stage	Left ventricular hypertrophy; Diastolic dysfunction; Increase in left atrial filling pressure
Advanced stage	Aggravated diastolic dysfunction; Extensive myocardial fibrosis Systolic dysfunction; Left ventricular dilatation

**Table 2:** Summary of cardioprotective effects exerted by different types and classes of flavonoids.

Flavonoid Class	Flavonoid Subclass	Study Design	Dose	Results	Reference
Anthocyanin	Anthocyanin	In vivo; T1DM rats	250 mg/kg/day	Improve cardiac dysfunction, significant reduction in cardiac hypertrophy and fibrosis.	Chen et al. 2016 [68]
Epigallocatechin-3-gallate	Flavanol	In vivo; Goto-Kakizaki rats	100 mg/kg/day	Improved cardiac mitochondrial function.	Liu et al. 2014 [59]
	Flavanol	In vivo; T1DM rats	2 mg/kg	Ameliorated diabetic phenotypes prevented cardiac injury, improved cardiac oxidative stress, and prevented cardiomyocyte apoptosis.	Othman et al. 2017 [69]
Epicatechin-rich (polyphenol)	Flavanol	In vivo; T2DM rats	100 mg/day	Improved cardiac mitochondrial function and structure in diabetic rats.	Taub et al. 2011 [70]
Luteolin	Flavone	In vivo & in vitro; H9C2 cells & T1DM mice	5, 10 µM; 20 mg/kg	Significantly reduced cardiac inflammation and oxidative stress via inhibition of the NF-κB pathway as well as increasing Nrf2 expression.	Li et al. 2016 [71]
	Flavone	In vivo; T1DM rats	100 mg/kg/day	Significantly improved cardiac function and mitochondrial function by increasing cardiac antioxidants.	Xiao et al. 2019 [72]
Baicalein	Flavone	In vivo; T1DM rats	200 mg/kg	Improved cardiac injury by increasing cardiac antioxidant levels; prevented cardiomyocyte inflammation and apoptosis by via inhibition of P13K/Akt pathway.	Ma et al. 2018 [73]
Fortunellin	Flavone	In vivo & in vitro; H9C2 cells & diabetic mice	10, 20 and 30 mg/kg; 20, 40 & 80 uM	Significantly attenuated cardiac structural and functional alterations by modulating cardiac inflammation and oxidative stress via the NF-κB pathway.	Zhao et al. 2017 [74]
Rutin	Flavone	In vivo; T1DM rats	50 mg/kg	Improved diabetic phenotypes; protected the heart against left ventricular hypertrophy and myocardial dysfunction.	Guimaraes et al. 2015 [75]
Diosmin	Flavone	In vivo; T1DM rats	100 mg/kg	Improved hyperglycemia and blood pressure as well protected the heart against cardiac injury, alleviated cardiac inflammation by downregulating pro-inflammatory cytokines, and ameliorated cardiac apoptosis.	Ali et al. 2019 [76]
Nobiletin	Flavone	In vivo; T1DM mice	50 mg/kg	Ameliorated cardiac dysfunction by improving cardiac oxidative stress via the inhibition of NADPH oxidase expression. Additionally, inhibited activation of c-Jun NH2-terminal kinase (JNK), P38, and NF-κB in the cardiac tissue.	Zhang et al. 2016 [77]
Kaempferol	Flavonol	In vivo; T1DM rats	20 mg/kg	Improved hyperglycemia condition; reduced cardiac injury and increased cardiac oxidative stress status via the upregulation of Nrf2.	Zhang et al. 2019 [78]
	Flavonol	In vivo; T1DM rats	20 mg/kg	Improved hyperglycemia; suppressed AGE-RAGE activation and suppressed PKC-MAPK pathways in inducing cardiac inflammation and oxidative stress.	Suchal et al. 2017 [79]
	Flavonol	In vivo; T1DM mice	100 mg/kg	Significantly attenuated cardiac hypertrophy by inhibiting MAPK pathways.	Feng et al. 2017 [80]
Quercetin	Flavonol	In vivo; T1DM rats	10, 25 and 50 mg/kg/bw	Protected against cardiac injury and increased cardiac antioxidants levels.	Roslan et al. 2016 [81]
	Flavonol	In vivo; high-cholesterol fed, hyperglycemic rats	0.5% *w*/*w* in rat feed	Improved diabetic phenotype and cardiac oxidative stress by upregulating Nrf2 expression.	Castillo et al. 2018 [82]
Fisetin	Flavonol	In vivo; T1DM rats	2.5 mg/kg	Improved diabetic phenotypes by alleviating hyperglycemia and hyperlipidemia; reduced cardiac injury and regressed cardiac function by suppressing oxidative stress, inflammation and apoptosis in the diabetic hearts.	Althunibat et al. 2019 [83]
Myricitrin	Flavonol	In vivo & in vitro; H9c2 cells & T1DM rats	300 mg/kg/day	Reduced AGE-induced cardiac inflammation and upregulated Nrf2 in cardiac tissue to improve oxidative stress status.	Zhang et al. 2017 [84]
Genistein	Isoflavone	In vivo; T1DM rats	10 and 50 mg/kg	Significantly improved cardiac function and alleviated cardiac oxidative stress by regulating the Nrf2/HO-1 pathway.	Jia et al. 2019 [85]

## Data Availability

No new data were created or analyzed in this study. Data sharing is not applicable to this article.

## Abbreviations

DCM	Diabetic cardiomyopathy
DPP-4	Dipeptidyl peptidase-4
GLP-1	Glucagon-like peptide-1
SGLT2	Sodium-dependent glucose transporter-2
AE	Aglycone equivalent
ESC	European Society of Cardiology
ROS	Reactive oxygen species
AGE	Advanced glycation end products
PKC	Protein kinase C
DAG	Diacylglycerol
NADPH	Nicotinamide adenine dinucleotide phosphate
MAPK	Mitogen-activated protein kinase
ERK	Extracellular-regulated kinase
JNK	c-Jun N-terminal kinase
SOD	Superoxide dismutase
H_2_O_2_	Hydrogen peroxide
RAGE	Receptor for AGE
Keap1	Kelch-like ECH-associated protein
IL	Interleukin
SIRT-1	Sirtuin-1
TLR-4	Toll-like receptor 4
TGF-β	Tumor growth factor β
Nrf2	Nuclear erythroid 2-related factor 2
CTGF	Connective tissue growth factor
iNOS	Inducible nitric oxide synthase
TNF-α	Tumor necrosis factor-α
ASK1	Apoptosis signal-regulating kinase 1
FOXO1	Forkhead box protein O1
NQO1	NADPH dehydrogenase
MnSOD	Manganese SOD
HO-1	Heme oxygenase-1
SGLT1	Sodium-dependent glucose transporter 1
AMPK-mTOR	AMP-activated serine/threonine protein kinase- mammalian target-of-rapamycin
